# Mobility anisotropy in the herringbone structure of asymmetric Ph-BTBT-10 in solution sheared thin film transistors†

**DOI:** 10.1039/d1tc01288f

**Published:** 2021-05-15

**Authors:** Adrián Tamayo, Sebastian Hofer, Tommaso Salzillo, Christian Ruzié, Guillaume Schweicher, Roland Resel, Marta Mas-Torrent

**Affiliations:** Institut de Ciència de Materials de Barcelona, ICMAB-CSIC, Campus de la UAB 08193 Bellaterra Spain mmas@icmab.es; Institute of Solid State Physics, Graz University of Technology Petersgasse 16 Graz 8010 Austria roland.resel@tugraz.at; Laboratoire de Chimie des Polymères, Faculté des Sciences, Université Libre de Bruxelles (ULB), Boulevard du Triomphe 1050 Brussels Belgium

## Abstract

Thin films of the organic semiconductor Ph-BTBT-10 and blends of this material with polystyrene have been deposited by a solution shearing technique at low (1 mm s^−1^) and high (10 mm s^−1^) coating velocities and implemented in organic field-effect transistors. Combined X-ray diffraction and electrical characterisation studies prove that the films coated at low speed are significantly anisotropic. The highest mobility is found along the coating direction, which corresponds to the crystallographic *a*-axis. In contrast, at high coating speed the films are crystallographically less ordered but with better thin film homogeneity and exhibit isotropic electrical characteristics. Best mobilities are found in films prepared at high coating speeds with the blended semiconductor. This work demonstrates the interplay between the crystal packing and thin film morphology and uniformity and their impact on the device performance.

## Abstract 2

Thin films of Ph-BTBT-10 are deposited by solution shearing at low and high coating velocities. Films coated at low speed are anisotropic, while the high coating speed films are less ordered but more homogenous and electrically isotropic.

## Graphical abstract

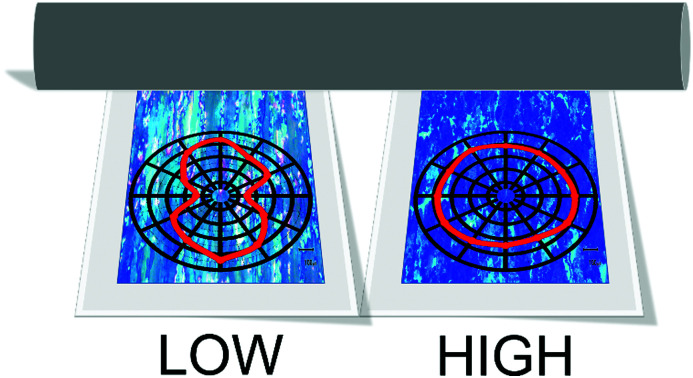

## Introduction

The transport properties of small molecule organic semiconductors are strongly ruled by their crystal structure. Molecular crystals are characterized by weak non-directional van der Waals intermolecular interactions and hence, molecules are prone to polymorphism.^1–3^ Many organic semiconductors such as rubrene,^4,5^ acenes,^2,6,7^ tetrathiafulvalenes^8–10^ and oligothiophenes^11,12^ have been reported to exhibit different polymorphs. Generally, the different polymorphs exhibit different charge transport behaviours when applied in organic field-effect transistors (OFETs) due to the differences in the overlap of the frontier orbitals (*i.e.*, charge transfer integrals).^13,14^ Additionally, the intrinsic anisotropy of organic crystals can also strongly influence the transport properties. Indeed, the different relative positions of neighbouring molecules in a crystal provides a variety of charge transfer integrals in the different crystallographic directions.^15–17^ Generally, the direction that displays the strongest electronic overlap between the π-orbitals of the organic semiconductor determines the path of the maximum charge transport, although other factors such as the charge-phonons interactions play also a crucial role.^18–20^ Noticeably, the crystal packing will strongly define the transport dimensionality and anisotropy in agreement with the directions where stronger intermolecular interactions are present. For instance, cofacial packings will tend to give one-dimensional (1D) electronic structures, whereas herringbone organisations will result in 2D structures.^21,22^

In organic semiconductor thin films, the conduction anisotropy is also closely connected to the morphological texture. Thin films are generally polycrystalline and depending on the experimental conditions employed to fabricate them, it is possible to prepare oriented crystal domains. While the charge transport in a polycrystalline film with randomly oriented domains will be isotropic, in a film with aligned crystallites, transport anisotropy will be expected accordingly to the molecular crystal packing.^23–28^ The crystal arrangement and the morphology of organic semiconductor thin films have been found to be dependent on the substrate,^6,29^ solvent and solution concentration,^30^ temperature^31^ and coating deposition parameters.^9,23,32–34^

The use of solution shearing deposition techniques has been shown to be a promising approach to obtain high performing thin film OFETs at low cost and on large areas. In these cases, often small molecule organic semiconductors are blended with a binding insulating polymer, such as polystyrene, to enhance the solution viscosity and favour the surface wetting and the formation of a homogenous film.^35^ Recent studies have been devoted to the understanding of the influence of the ink formulation, temperature and deposition speed on the final thin film properties.^24,33,36–39^ In particular, the latter parameter has an important impact on the crystallization and nucleation of the organic semiconductor, and hence, on its thin film morphology. At low speed, the crystallization occurs in the convective regime and oriented nearly single-crystalline films can be produced because the crystallization takes place at the meniscus contact line as the solvent is evaporated. However, at higher speeds, the crystallization takes place thanks to nucleation and coalescence from a supersaturated solution and therefore more randomly oriented crystals are formed.^9,40,41^

**Fig. 1:**
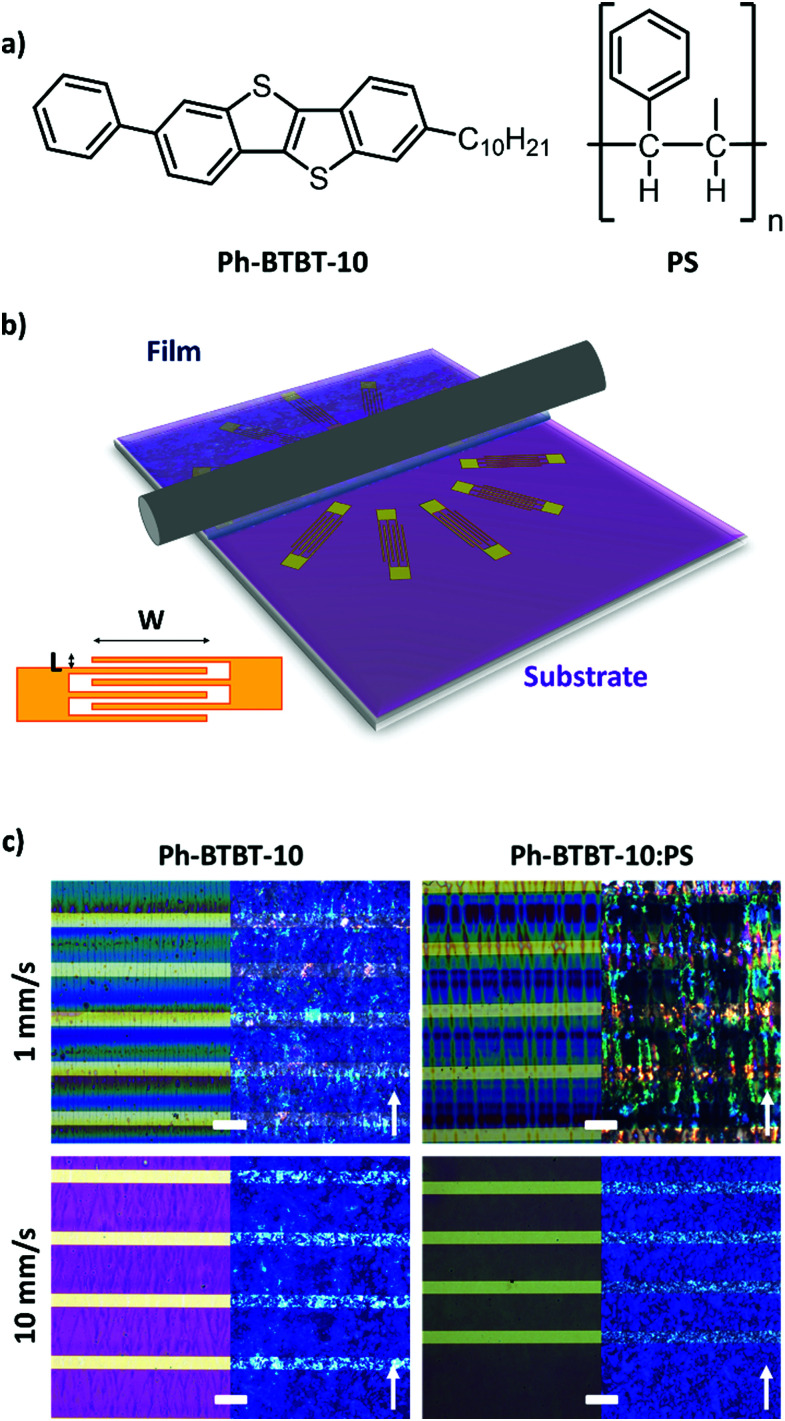
(a) Chemical structure of the molecule Ph-BTBT-10 and of polystyrene (PS). (b) Schematic illustration of the BAMS technique together with the used electrode layout. (c) Non-polarized (left) and polarized (right) microscopy images of the Ph-BTBT-10 thin films prepared from PhCl solutions on the interdigitated gold electrodes. Scale bar: 100 μm. The white arrow indicates the shearing direction.

Herein, we report on the influence of the solution shearing speed on the charge transport characteristics of thin film OFETs based on the asymmetric semiconductor 2-Decyl-7-phenyl[1]benzothieno[3,2-*b*][1]benzothiophene^31,42,43^ (Ph-BTBT-10, Fig. 1a) and blends of this molecule with polystyrene (PS). By combining in-depth X-ray diffraction studies and electrical transport characterization, we elucidate that at low coating speed the films crystallise with a preferential direction and a significant anisotropic transport is found despite the 2D herringbone packing of this semiconductor. However, electrically isotropic films are formed at high speed due to less ordered crystals. The optimum field-effect mobility is found with the blended films at high coating speed, which is attributed to the formation of more homogenous thin films.

## Results and discussion

Ph-BTBT-10 has already been reported to display excellent performance as active material in single crystal and thin film OFETs.^31,42^ Nevertheless, so far a post-processing thermal annealing has been reported to be essential to reach the thermodynamic bulk polymorph, also called bilayer structure, to reach a higher field-effect mobility.

In this work, thin films of Ph-BTBT-10 and blends of Ph-BTBT-10:PS were prepared by the Bar-Assisted Meniscus Shearing (BAMS) technique (Fig. 1b). This technique has previously been shown to lead to highly crystalline thin films with high throughput.^44,45^ The use of polymeric blends in thin films prepared by BAMS has also been demonstrated to facilitate the processing of small molecule semiconductors and to promote an enhanced thin film crystallinity and electrical performance of the devices. Additionally, the vertical stratification that takes place during the film deposition typically results in the formation of a bottom polymeric layer that passivates the SiO_*x*_ reducing the presence of charge traps.^44–48^ After an optimisation process of the ink formulation where different solvents, PS polymer weights and Ph-BTBT-10 : PS ratios were tested (Table S1, ESI†), the conditions selected were 2.0% w/w chlorobenzene (PhCl) and 2.5% w/w *o*-xylene based solutions and, in the case of blends, 280 kDa PS was chosen with a ratio Ph-BTBT-10 : PS 2 : 1. In all the cases, the films were deposited at high temperature (*i.e.*, 105 °C) without performing a post-annealing step which could affect the morphology and structure of the as-prepared films. To study the influence of the coating speed, the films were deposited at 1 and 10 mm s^−1^, which correspond to the capillary regime condition and the transition speed towards the viscous drag regime, respectively, in the BAMS technique.^9^

The polarized optical microscopy (POM) images of the films prepared from PhCl and *o*-xylene are shown in Fig. 1c and Fig. S1, ESI,† respectively. In both cases, the films deposited at low shearing speed (1 mm s^−1^) appear as well-oriented small stripe-shaped crystals aligned along the shearing direction. In contrast, we observe that the films prepared at high shearing speed (10 mm s^−1^) show a more uniform coverage without a clear preferential orientation. The only distinguishable feature between the films based on pristine Ph-BTBT-10 and Ph-BTBT-10:PS blends was the domain size, which was slightly larger for the pristine Ph-BTBT-10 film.

**Fig. 2:**
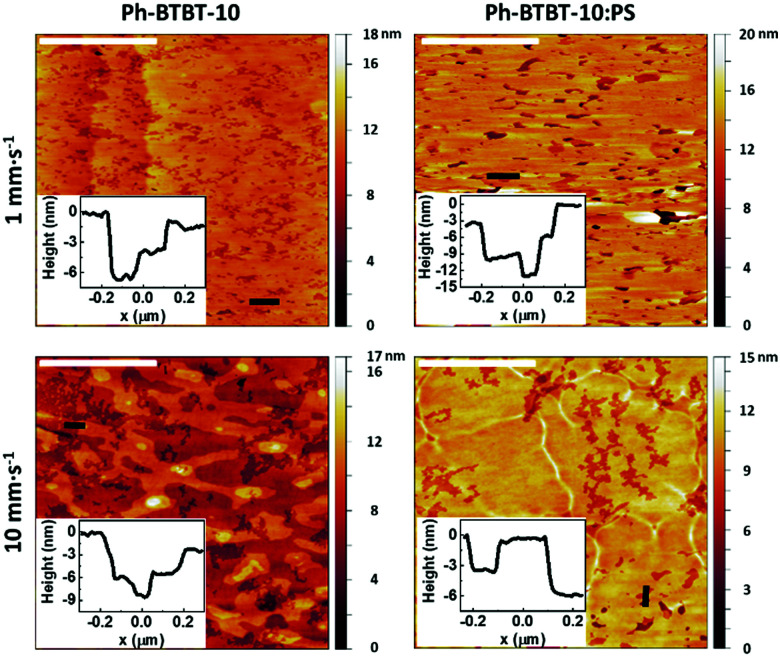
AFM topography images of Ph-BTBT-10 and Ph-BTBT-10:PS thin films deposited by BAMS using PhCl as solvent, at low and high coating speed. Scale bar: 2 μm. The insets are the high profiles along the black lines marked in the images.

The films were also characterized by atomic force microscopy (AFM) in order to determine the nanomorphology of the thin film surface (Fig. 2 and Fig S2, ESI†). All the films show smooth mesoscopic areas, which exhibit a similar nanostructure. Analysing the films profile, we can observe the step edges of around 2.9 ± 0.3 and 5.8 ± 0.4 nm high, in agreement with Ph-BTBT-10 mono- and bilayers (the length of the molecule is ∼2.6 nm), as it was previously reported by Iino *et al.*^31,49^ Generally, the mesoscopic areas appear more uncompleted when the films are prepared at high speed, especially when the binding PS polymer is not used.

**Fig. 3:**
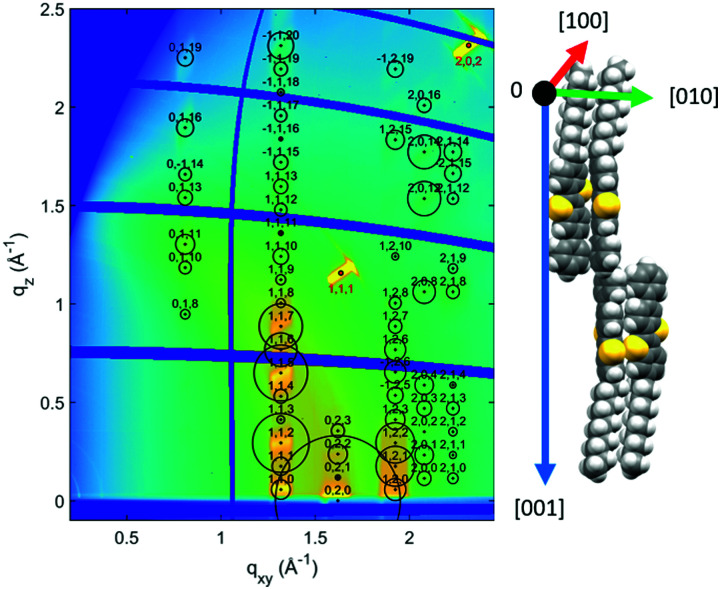
Reciprocal space map of a Ph-BTBT-10 thin film prepared by BAMS from a PhCl solution without added PS using a coating velocity of 1 mm s^−1^ (left). Black markers designate peak positions of the known crystal structure, the area of the circles is proportional to the structure factor (square root of intensity) of the peaks. Red markers indicate peaks from the silicon substrate. Packing motif of the molecules relative to the crystallographic unit cell directions (right).

The crystallographic phase present within the films was identified by grazing incidence X-ray diffraction (GIXD) investigations. The result from a representative sample deposited from PhCl a solution is shown in Fig. 3 (see Fig. S3, ESI,† for films prepared from *o*-xylene). The intensity distribution of the Bragg peaks within the reciprocal space map is compared to calculated structure factors of the known bulk crystal structure of Ph-BTBT-10.^22^ A good agreement is found with the theoretically expected peak intensities, confirming the formation of the bulk phase of Ph-BTBT-10. Additionally, these GIXD investigations reveal that the crystallites show a preferred orientation with the crystallographic (001) plane parallel to the substrate surface. In terms of molecular packing, this means that the herringbone layers-formed by the aromatic BTBT molecular cores - are parallel to the substrate surface.

**Fig. 4:**
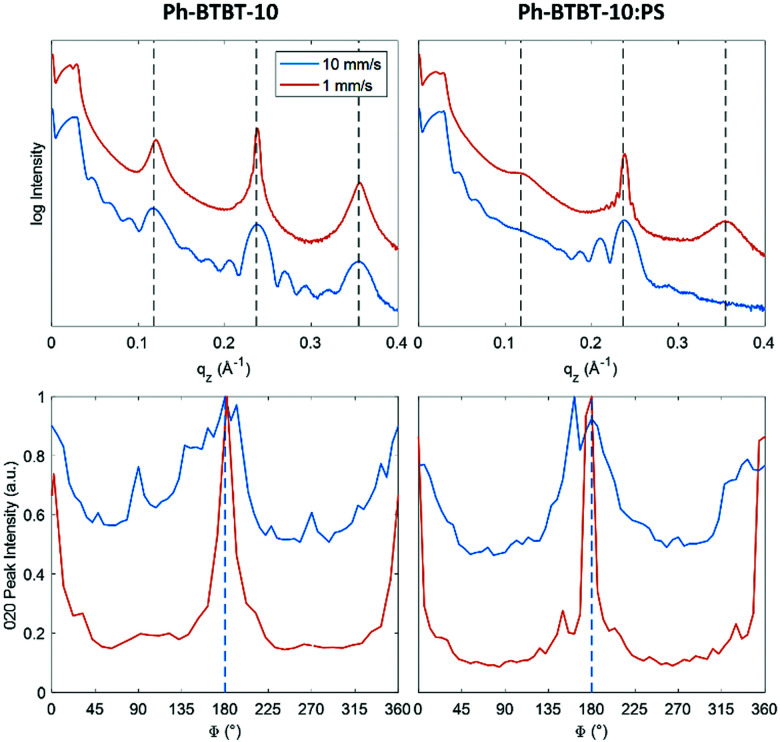
X-Ray reflectivity curves of BAMS coated Ph-BTBT-10 thin films prepared from PhCl solution without (left) and with (right) PS binding polymer (top) and rotation-dependent intensity of the 0 ± 20-peaks from GIXD with respect to the coating direction indicated by the dashed line (bottom) for different coating speeds.

Fig. 4(top) show the specular X-ray reflectivity (XRR) curves for BAMS coated films, prepared from PhCl solutions, with and without added PS. Corresponding data for samples prepared from o-xylene solvent can be found in the ESI† (Fig. S4). The XRR curves show a significant dependence on the coating speed. Faster coated samples show Kiessig fringes in the range of *q* = 0.02 Å^−1^ to 0.08 Å^−1^, indicating the formation of layers with constant thicknesses. A characteristic layer thickness of 25 nm was determined. The slower coated samples do not show Kiessig fringes, which is a sign of thin films with high surface roughness. Furthermore, diffraction features are visible at *q*_*z*_ = 0.12 Å^−1^, 0.24 Å^−1^ and 0.36 Å^−1^ which represent the first three orders of the 00L peak series. The alternating broadening of the 00L diffraction peaks can be explained by defects within the crystal structure.^50^ The coating speed has a strong influence on the width of the diffraction peaks. The peak widths (Δ*q*_*z*_) of the 00 ± 2 Bragg peaks were determined and analysed. We found values of around 0.0057 Å^−1^ (0.0045 Å^−1^ without PS) for the low coating speed samples, while a considerably larger peak width of 0.0170 Å^−1^ (0.0185 Å^−1^ without PS) is found for films prepared by high coating speeds. The peak width can be related to the vertical crystal size (Table 1), values of 110 nm and 37 nm are found for the films based on blends deposited at low and high coating speeds, respectively. At low coating speeds, oscillations are observed around the 00L Bragg peaks. Such Laue oscillations are a consequence of homogenous vertical size of the crystallites. Combining the results from the Kiessig fringes (thickness 25 nm) with the estimated length of the crystals (vertical size of 37 nm, 34 nm without PS) reveal that in case of large coating speeds the layers show constant film thickness with continuous crystalline character.

**Table 1:** Summary of vertical crystal size from the width of the 002 Bragg peak and in-plane mosaicity of Ph-BTBT-10 transistor samples as a function of coating speed and addition of polystyrene

Sample	Crystal size [nm]	In-plane alignment FHWM [deg]
1 mm s^−1^ PS	110	9.8
10 mm s^−1^ PS	34	36
1mm s^−1^ without PS	140	8.4
10 mm s^−1^ without PS	37	42

For analysing the in-plane alignment of the Ph-BTBT-10 crystallites, the rotation (*φ*) dependence of the 0 ± 20 peak intensities were extracted from the rotated GIXD measurements (Fig. 4 (bottom)). Two peaks can be detected, corresponding to a maximum intensity of the 0 ± 20 peak at *φ* = 0° and 180° (*i.e.*, with the primary X-ray beam parallel to the coating direction). As the scattering vector is about perpendicular to the primary X-ray beam for the 0 ± 20 peak and diffraction occurs from crystallographic planes, where the surface normal vector has an equal direction to the scattering vector. This means for the present case that the crystals are aligned with the [010] direction perpendicular and the [100] direction parallel to the shearing direction. Additionally, these data are used to determine the influence of the coating speed on the in-plane alignment of the crystallites. For this analysis the widths (full width at half maximum) were used. Values of 9.8° and 8.4° were found at low coating speeds (Table 1), for samples with and without PS, respectively. At high coating speeds, the diffraction intensity of the 020 peak is present by a dominating constant contribution together with peaks of considerably larger peak widths of 36° and 42°, again for samples prepared with/without addition of PS. Therefore, it can be concluded that the samples prepared at the low coating speed of 1 mm s^−1^ show a considerably higher degree of crystal alignment (and thereby a smaller in-plane mosaicity) than the samples prepared at the high coating speed of 10 mm s^−1^.

**Fig. 5:**
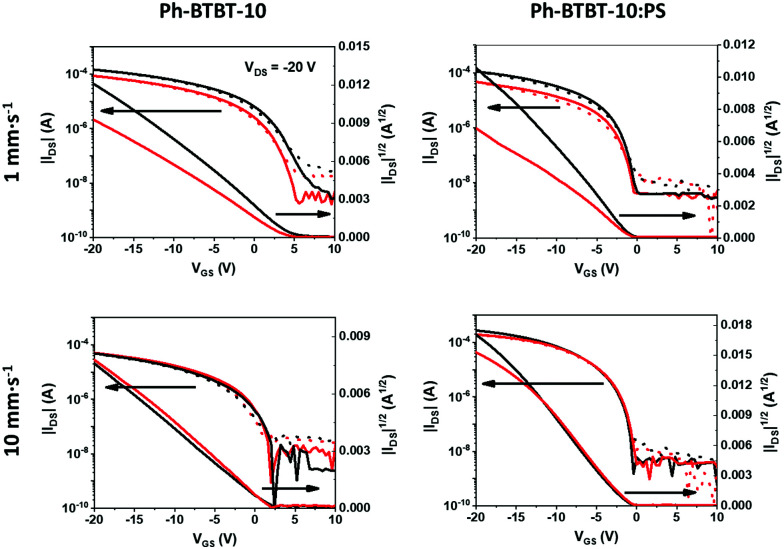
Transfer characteristics in the saturation regime (*V*_DS_ = −20 V) of typical Ph-BTBT-10 and Ph-BTBT-10:PS films prepared by BAMS from PhCl solutions at 1 and 10 mm s^−1^. Continuous lines correspond to forward, while dotted lines correspond to reverse sweeps of gate voltages. Black lines correspond to devices with the channel length (*L*) parallel to the coating direction and red lines correspond to the perpendicular ones.

All the films were electrically characterized as active layers in OFETs under ambient conditions. Fig. 5 shows the transfer characteristics in the saturation regime for the devices based on thin films of Ph-BTBT-10 and Ph-BTBT-10:PS deposited from PhCl solutions at high and low coating speeds. In these plots the electrical characteristics of the devices measured with the channel length (*L*), that is the direction where the charge transport takes place, perpendicular (red) and parallel (black) to the coating direction are included. The corresponding output characteristics are shown in Fig. S5 (ESI†) and the electrical characteristics of devices prepared from o-xylene are displayed in Fig. S6 and S7 (ESI†). All the devices exhibit p-type OFET characteristics with low hysteresis. In general, the devices based on films of Ph-BTBT-10 reveal threshold voltages (*V*_TH_) positively shifted with respect to the ones based on the semiconductor blend (see Table S1, ESI†). This can be indicative of some unintentional doping, probably caused by the presence of H_2_O on the SiO_*x*_ surface. This effect is commonly found in OFETs based on organic semiconductors processed in environmental conditions. Regarding the electrical anisotropy, we find that while no appreciable differences between the parallel and perpendicular devices are observed in the films coated at high speed, clearly the films coated at low speed display distinct characteristics depending on the measurement direction. In fact, the devices with *L* parallel with the solution shearing direction exhibit a higher source-drain current (*I*_SD_). Such observed anisotropy at low coating speed is in agreement with the fact that aligned crystals are formed in these conditions.

**Fig. 6:**
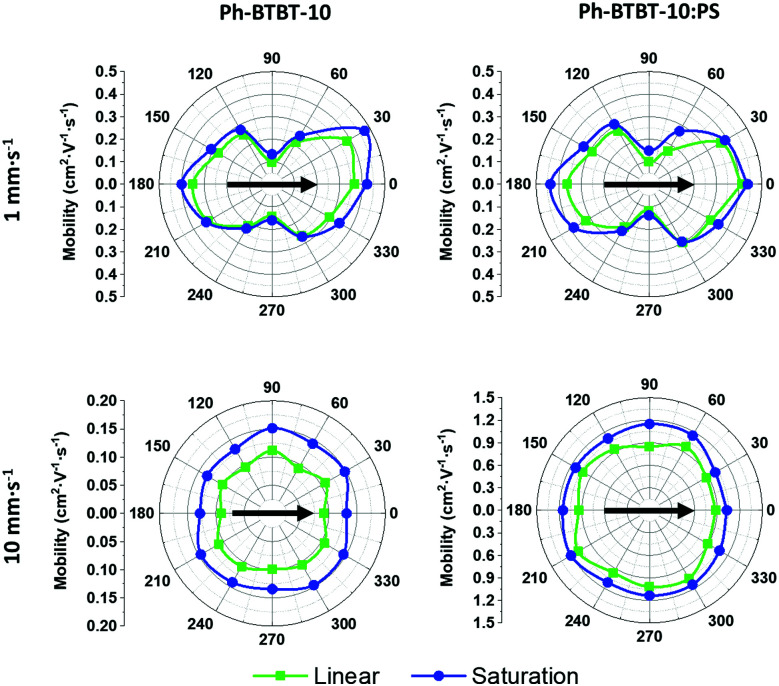
Polar plot of the linear and saturation mobility of the Ph-BTBT-10 and Ph-BTBT-10:PS films prepared from PhCl solutions at low and high coating speed. The angle corresponds to the conducting channel L with respect to the coating direction. The direction of the solution shearing is indicated with a black arrow. The channel length of these devices was 100 μm.

In order to better evaluate the field-effect mobility of the devices along different thin film directions, OFETs with electrodes with star-oriented layout were fabricated (see Fig. 1b). In Fig. 6 (and Fig. S8 (ESI†) for devices deposited from *o*-xylene) the mobility of the devices is plotted as a function of the angle between *L* (*i.e.*, charge-transport path) and the coating direction. At low coating speed, the highest mobility is found when the device channel *L* is parallel with respect to the coating direction, achieving a mobility of 0.48 and 0.43 cm^2^ V^−1^ s^−1^ for Ph-BTBT-10 and Ph-BTBT-10:PS films, respectively. On the other hand, the lowest mobility value was found to be 0.14 cm^2^ V^−1^ s^−1^ for both films in the perpendicular direction. Thus, the anisotropy ratio was estimated to be in the range 3.3–3.7 for Ph-BTBT-10 films and 2.9–3.2 for the Ph-BTBT-10:PS ones. Taking into account the X-ray results, we can affirm that the maximum mobility is thus achieved along the *a*-axis of the crystal structure. This is fully in accordance with the theoretical work recently reported by Baggioli *et al.*^51^ In this work the authors show that within the herringbone plane, the maximum mobility is found along the *a*-axis with an anisotropy ratio of around 2. The higher anisotropy ratio found here experimentally can be due to the influence of the intergrain boundaries.

Considering the devices deposited at high shearing speed, a quasi-ideal electrical isotropy was found (ratio *μ*_‖_/*μ*_⊥_ ∼ 1). Thin films of Ph-BTBT-10:PS displayed the highest mobility of 1.46 cm^2^ V^−1^ s^−1^ (1.3 ± 0.1 cm^2^ V^−1^ s^−1^ on average), while the mobility of the films based on only the organic semiconductor was 0.22 cm^2^ V^−1^ s^−1^ (0.16 ± 0.05 cm^2^ V^−1^ s^−1^ on average) (see also Fig. S9, ESI†). It is worth noticing that the use of blends is significantly enhancing the device mobility of the films deposited at high coating speed to almost one order of magnitude. It is known that the viscosity of the ink solutions promotes the formation of more homogenous films, which is particularly important when the films are deposited at high shearing speed. Remarkably, the high isotropic mobility achieved with the blends at high speed is between 2 and 3 times higher than the best one found for the preferentially aligned crystallites obtained at low coating speed. This implies that not only the intermolecular interactions are playing a role but also the thin film morphology. This agrees with the XRR results which indicated that the films coated by high speed are homogenous with a constant film thickness.

The main difference observed between the films prepared from PhCl or *o*-xylene was the electrical isotropy/anisotropy. At low shearing speed, *o*-xylene films reveal a lower anisotropy ratio of 1.7–2.0 for Ph-BTBT-10 films and 2.0–2.3 for the Ph-BTBT-10:PS ones. Further, in the films prepared at high speed a small anisotropy ratio was found in the range 1.1–1.3 for both Ph-BTBT-10 and Ph-BTBT-10:PS films. Therefore, the ideal isotropy behaviour was only possible to obtain using PhCl as solvent.

## Experimental

### Solution preparation

Ph-BTBT-10 and PS 10 000 g mol^−1^ and 280 000 g mol^−1^ (PS10 and PS280, respectively) were purchased from TCI Chemical Industry and Sigma-Aldrich, respectively, and used without further purification. Solutions of Ph-BTBT-10 and Ph-BTBT-10:PS were prepared in PhCl and *o*-xylene with a final concentration of 2.0 and 2.5% w/w, respectively. Before to solution deposition, the solutions were heated at the substrate temperature used for the coating process.

### Materials and device fabrication

Interdigitated electrodes were patterned by photolithography on heavily n-doped Si wafer (Si-Mat) with a 200 nm thick layer of SiO_*x*_. Subsequently, a 5 nm of Cr (acting as adhesion layer) followed by 40 nm of Au were deposited by thermal evaporation. The channel lengths (*L*) varied from 25 to 200 μm and the channel width/length ratio was always set constant to 100. Substrates were cleaned by sonication in HPLC grade acetone and isopropanol and then dried under nitrogen. The gold electrodes were chemically modified with a self-assembled monolayer of 2,3,4,5,6-Pentafluorothiophenol (PFBT), which has been proved to improve the charge injection from the source/drain electrodes to the organic semiconductor.^44^ For this, the gold electrodes were first activated with an ultraviolet ozone generator for 25 min and then immersing in a 15 mM solution of PFBT in isopropanol for 15 min. Finally, the substrates were washed with pure isopropanol to remove the PFBT excess. The Ph-BTBT-10 films were then deposited by Bar-Assisted Meniscus Shearing (BAMS) as previously reported.^50^ The solution shearing deposition was carried out at a substrate temperature of 105 °C and with coating speed of 1 and 10 mm s^−1^. Note that all the fabrication process was carried out under ambient conditions and no post-thermal treatments were required.

### Thin-film characterization

The optical microscopy images were taken using an Olympus BX51 equipped with polarizer and analyser. Surface topographies on the devices were examined by a 5500LS SPM system from Agilent Technologies and subsequent data analysis was performed by using Gwyddion 2.56 software.

X-Ray Reflectivity (XRR) measurements were carried out with a PANalytical Empyrean diffractometer in *θ*–*θ* geometry using CuKα radiation. On the incident side a parallel beam X-ray mirror was used for monochromatization. At the diffracted beam path an anti-scatter slit as well as a 0.02 rad Soller slit was used together with a PIXcel3D detector operating in receiving 0D-mode. The data is converted into reciprocal space by 
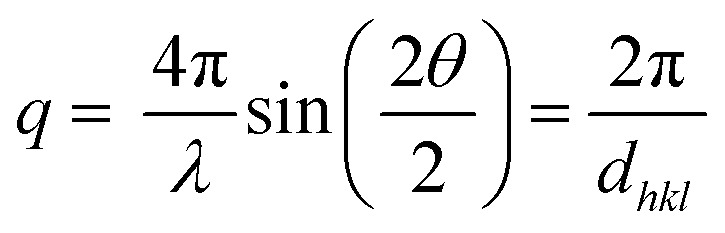
 with *λ* as the wavelength of the primary X-ray beam, 2*θ* the scattering angle and *d*_*hkl*_ the interplanar distance of the (*hkl*) plane. Samples without gold electrodes were also investigated. Crystal sizes was determined by the Scherrer equation using 
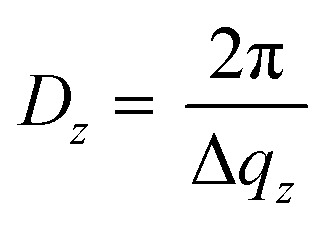
 with *D*_*z*_ as the vertical crystal size and Δ*q*_*z*_ as the peak width.

Rotated grazing incidence X-Ray diffraction (GIXD) was measured at the beamline XRD1 at Elettra Synchrotron Trieste with an X-ray radiation wavelength of 1.40 Å using an incidence angles, varied between *α*_*i*_ = 0.2° up to 3.0° on a goniometer in Kappa geometry.^52^ A *φ* – rotation of the samples was performed around the substrate surface normal. Care was taken to place every sample of the series with the coating direction along the X-ray beam, defining a defined azimuthal alignment of the samples (*φ* = 0°). The sample rotation was carried out with a step width of 6° integrating the intensity for 5 s for each image. A PILATUS 2M detector was used to collect the diffracted intensity. The experimental data were evaluated with the in-house developed software GIDVis.^53^ The components of the scattering vector (*q*_*x*_, *q*_*y*_, *q*_*z*_) scattering vector components are determined for each detector pixel from the incident angle *α*_*i*_ and the outgoing angle *α*_f_ in the sample coordinate system by using a calibration measurement from an LaB_6_ film. Data from GIXD measurements are presented as reciprocal space maps by summing up the intensities of a full rotation of 360°. Finally, the intensity is plotted as a function of the out-of-plane (*q*_*z*_) and in-plane component (*q*_*xy*_) of the scatting vector with *q*_*xy*_^2^ = *q*_*x*_^2^ + *q*_*y*_^2^. A second method is used for presenting the GIXD data collected from samples with transistor structures. The intensities of a selected area around the 020 peak of each individual reciprocal space maps are integrated and plotted as a function of the rotation angle *φ*.

### Device characterization

The transfer and output characteristics of the devices were measured with an Agilent B1500A semiconductor device analyser connected to the samples with a Karl SÜSS probe station, at ambient conditions. The devices were carefully isolated by scratching the films before performing the measurements. The characteristic field-effect mobility (*μ*) and threshold voltage (*V*_TH_) parameters of the transistors were extracted in the linear and saturation regime using the following classic MOSFETs equations:1
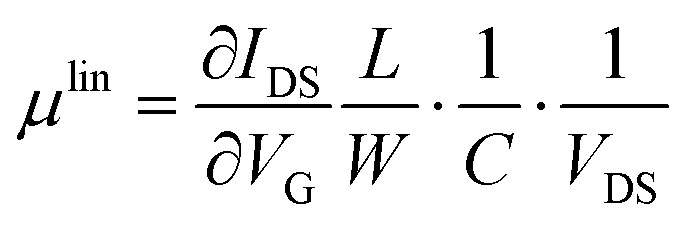
2
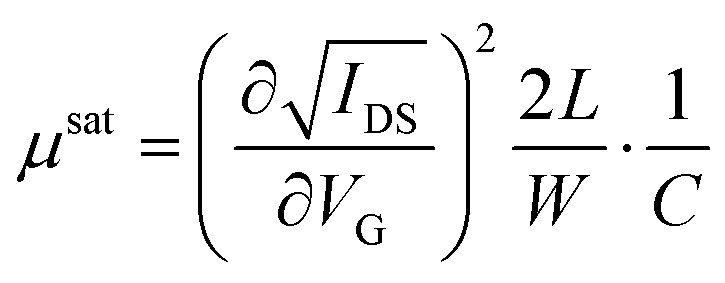
where *W* and *L* are the width and length of the channel, respectively, *C* is capacitance per unit area of the dielectric (*C* = 17.26 nF cm^−2^), *I*_DS_ the measured source-drain current, *V*_DS_ the source-drain current and *V*_G_ the applied gate voltage. For each condition and measurement direction, the devices parameters were extracted from at least 40 devices from 2 substrates to ensure thin film homogeneity and reproducibility.

## Conclusions

To sum up, thin films of the organic semiconductor Ph-BTBT-10 and blends of it with PS have been prepared by a solution shearing technique at two deposition speeds: 1 mm s^−1^ (low) and 10 mm s^−1^ (high). Chlorobenzene as well as *o*-xylene were used as solvents. In all the cases, the organic semiconductor crystallised in the bulk phase following a 2D herringbone arrangement. Further, all the films exhibited a p-type field-effect behaviour when implemented in OFETs. However, clear differences in the performance were observed due to influence of the coating speed on the crystallization and thin film morphology/uniformity.

At low coating speed, films grow with the *a*-axis aligned with the coating direction, where the maximum field-effect mobility is found. The thin film electrical anisotropy in the films is in the range 3–4 for PhCl and 1.5–2.5 for *o*-xylene. This is larger than what was theoretically expected for a single crystal (especially in the films without PS) which can be explained by the impact of the intergrains. In sharp contrast, the crystals in the films coated at high speed are not aligned in the *ab* plane and, accordingly, they exhibit isotropic charge transport mobilities. Remarkably, the blended films reveal a high mobility of 1.3 cm^2^ V^−1^ s^−1^ and 1.0 cm^2^ V^−1^ s^−1^ when they were prepared from PhCl or *o*-xylene solutions, respectively.

This work elucidates that the coating speed has a strong impact on the thin film crystallisation and thin film morphology, both features play a crucial role in determining the final device performance.

## Author contributions

The manuscript was written through contributions of all authors. All authors have given approval to the final version of the manuscript.

## Conflicts of interest

The authors declare no conflict of interest.

## Supplementary Material

TC-009-D1TC01288F-s001
