# Supplementary material for: Mobility anisotropy in the herringbone structure of asymmetric Ph-BTBT-10 in solution sheared thin film transistors
Source: J Mater Chem C Mater. 2021 May 15;9(22):7186–93. doi: 10.1039/d1tc01288f (PMC8191576; doi:10.1039/d1tc01288f)
Supplement: TC-009-D1TC01288F-s001 [file TC-009-D1TC01288F-s001.pdf]

Electronic Supplementary Material (ESI) for Journal of Materials Chemistry C.
This journal is © The Royal Society of Chemistry 2021

SUPPORTING INFORMATION

Mobility anisotropy in the herringbone structure of asymmetric Ph-BTBT-10 in solution sheared thin film transistors

Adrián Tamayo,[a,‡] Sebastian Hofer,[b,‡] Tommaso Salzillo,[a,d] Christian Ruzié,[c] Guillaume Schweicher,[c] Roland Resel[b] * and Marta Mas-Torrent [a] *

[a] Institut de Ciència de Materials de Barcelona, ICMAB-CSIC, Campus de la UAB, 08193 Bellaterra, Spain. e-mail: mmas@icmab.es

[b] Institute of Solid State Physics, Graz University of Technology, Petersgasse 16, Graz 8010, Austria. e-mail: roland.resel@tugraz.at

[c] Laboratoire de Chimie des Polymères, Faculté des Sciences, Université Libre de Bruxelles (ULB), Boulevard du Triomphe 1050 Brussels, Belgium.

[d] Current address: Department of Chemical and Biological Physics, Weizmann Institute of Science, Herzl street 234, 76100 Rehovot, Israel

[‡] Equally contributed to this work.

The Supporting Information gives an overview on the comprehensive approach of the experimental work. Scheme 1 gives a sketch of the transistor geometries used in this work. Thin film transistors were fabricated by wide variation of the organic film preparation conditions. Five different solvents with and without blending of the Ph-BTBT-10 solution with polystyrene (with variation of blending ratios and molecular weight) were used; two different coating speeds were applied. The respective device performances are listed within **Table S1**. Based on these results we decided to perform detailed work on thin films prepared by solutions from chlorobenzene and from o-xylene.

The experimental results of both type of device series are discussed in the main paper. Due to limited space, only the results on chlorobenzene based system are depicted as figures in the main paper. The corresponding results on the systems prepared from o-xylene solutions are given here: **Figure S1** presents optical microscopy images with and without polarized light; **Figure S2** the mesoscopic morphology by AFM images; **Figure S3** a reciprocal space map of a representative sample calculated from GIXD experiments; **Figure S4** X-ray reflectivity (XRR) curves and φ-scans of the 0±20 peak; **Table S2** the observed peak widths of the specular diffraction peak measurements; **Figure S6** and **Figure S7** the transfer and output characteristics of the devices, respectively and **Figure S8** the orientation dependence of the charge transport mobility. The only result on chlorobenzene transistors within the Supporting Information are the output characteristics presented in **Figure S5**.

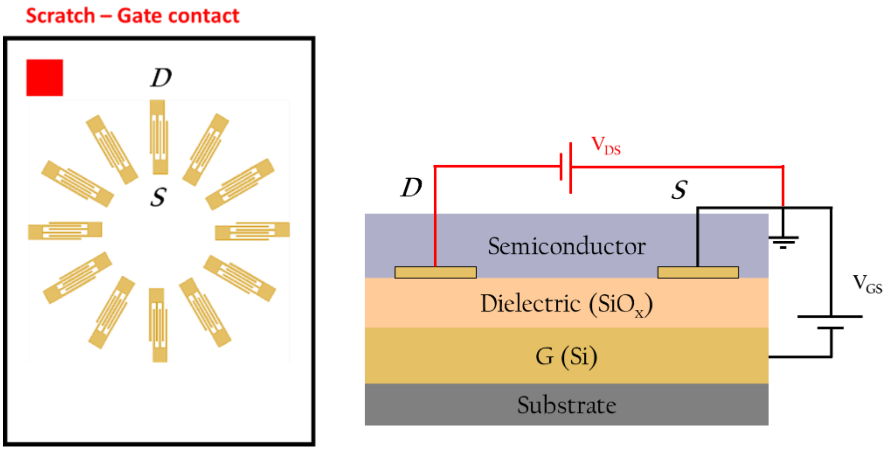

Scheme 1. OFET structure used in this work.

Table S1. Average of main OFET parameters measured and its standard deviation extracted from transfer characteristics for the fabricated devices. At least 42 devices were prepared for each specific experimental condition. The selected conditions are highlighted in bold.

			Coating speed: 1 mm/s		Coating speed: 10 mm/s	
Solvent	OSC:PS Ratio	PS Molecular Weight (kg·mol^{-1})	Mobility (cm^2·V^{-1}·s^{-1})	Threshold Voltage (V)	Mobility (cm^2·V^{-1}·s^{-1})	Threshold Voltage (V)
o-xylene	1:0	-	0.4 ± 0.2	4 ± 2	0.5 ± 0.2	2 ± 1
	4:1	10	0.4 ± 0.1	-1 ± 2	0.4 ± 0.2	0 ± 1.
	2:1	280	0.6 ± 0.3	-1.2 ± 0.8	1.0 ± 0.2	-0.7 ± 0.4
chlorobenzene	1:0	-	0.42 ± 0.09	2 ± 1	0.16 ± 0.05	0 ± 2
	4:1	10	1.03 ± 0.06	-1.9 ± 0.3	0.67 ± 0.07	-0.9 ± 0.2
	4:1	280	0.40 ± 0.08	-1.1 ± 0.2	0.9 ± 0.2	-0.8 ± 0.2
	2:1	280	0.4 ± 0.1	-1.5 ± 0.5	1.3 ± 0.07	-0.6 ± 0.3
	1:2	280	0.06 ± 0.05	-4 ± 2	0.04 ± 0.01	-10 ± 3
anisole	1:0	-	0.002 ± 0.001	2 ± 4	0.03 ± 0.01	0.4 ± 0.9
	4:1	10	0.04 ± 0.03	2 ± 2	0.9 ± 0.3	-1.1 ± 0.6
o-dichlorobenzene	1-0	-	0.23 ± 0.09	3 ± 1	0.012 ± 0.004	-9 ± 3
	4:1	10	0.31 ± 0.06	0.3 ± 0.9	0.32 ± 0.06	-1.1 ± 0.5
decalin	1:0	-	0.5 ± 0.2	7 ± 2	0.07 ± 0.03	8 ± 4
	4:1	10	0.3 ± 0.1	2 ± 3	0.35 ± 0.06	4 ± 3

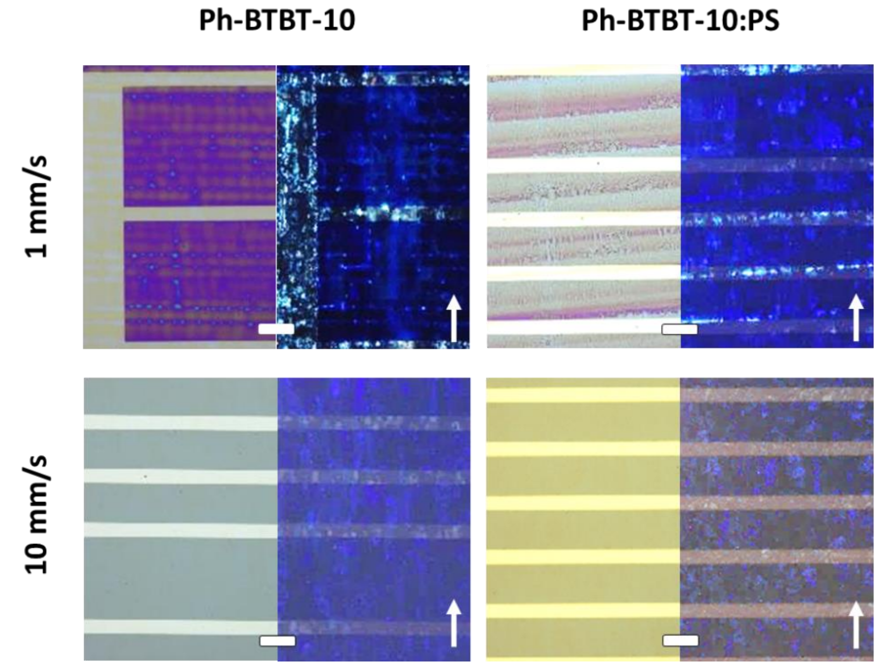

Fig. S1. Non-polarized (left) and polarized (right) microscopy images of the Ph-BTBT-10 thin films prepared from o-xylene. Scale bar: 100 μm. The white arrow indicates the shearing direction.

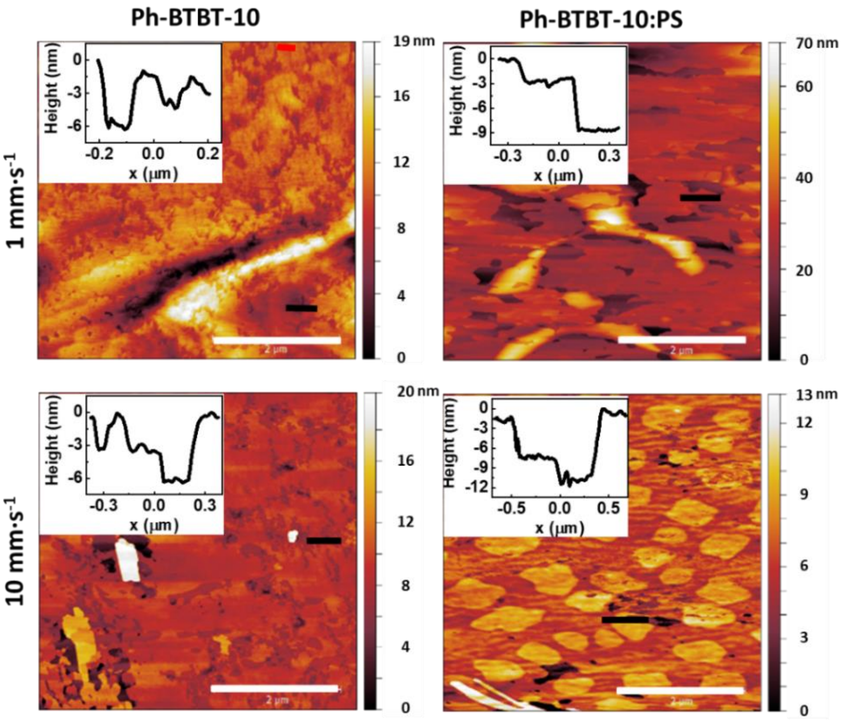

Fig. S2. AFM topography images of Ph-BTBT-10 and Ph-BTBT-10:PS blend thin films deposited by BAMS, using o-xylene as solvent, at low and high coating speed. Scale bar: 2 µm. Inset: high profiles extracted along the black line of the images.

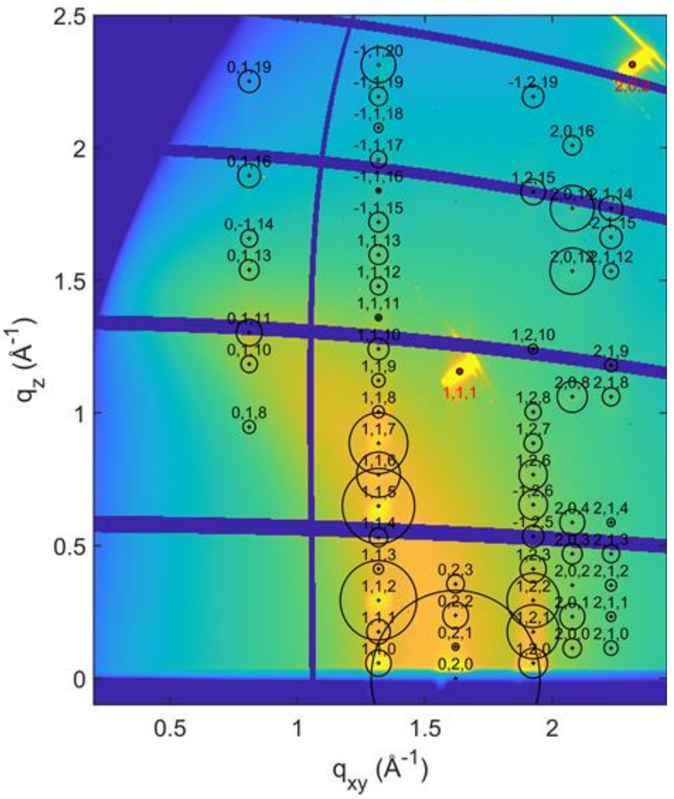

Fig. S3: Reciprocal space map of a Ph-BTBT-10 thin film prepared by bar assisted shear coating from o-xylene solution without added PS using a coating velocity of 1 mm/s. Black markers designate peak positions of the known crystal structure (bulk phase), the area of the circles is proportional to the structure factor of the peaks. Red markers indicate peaks from the Si substrate.

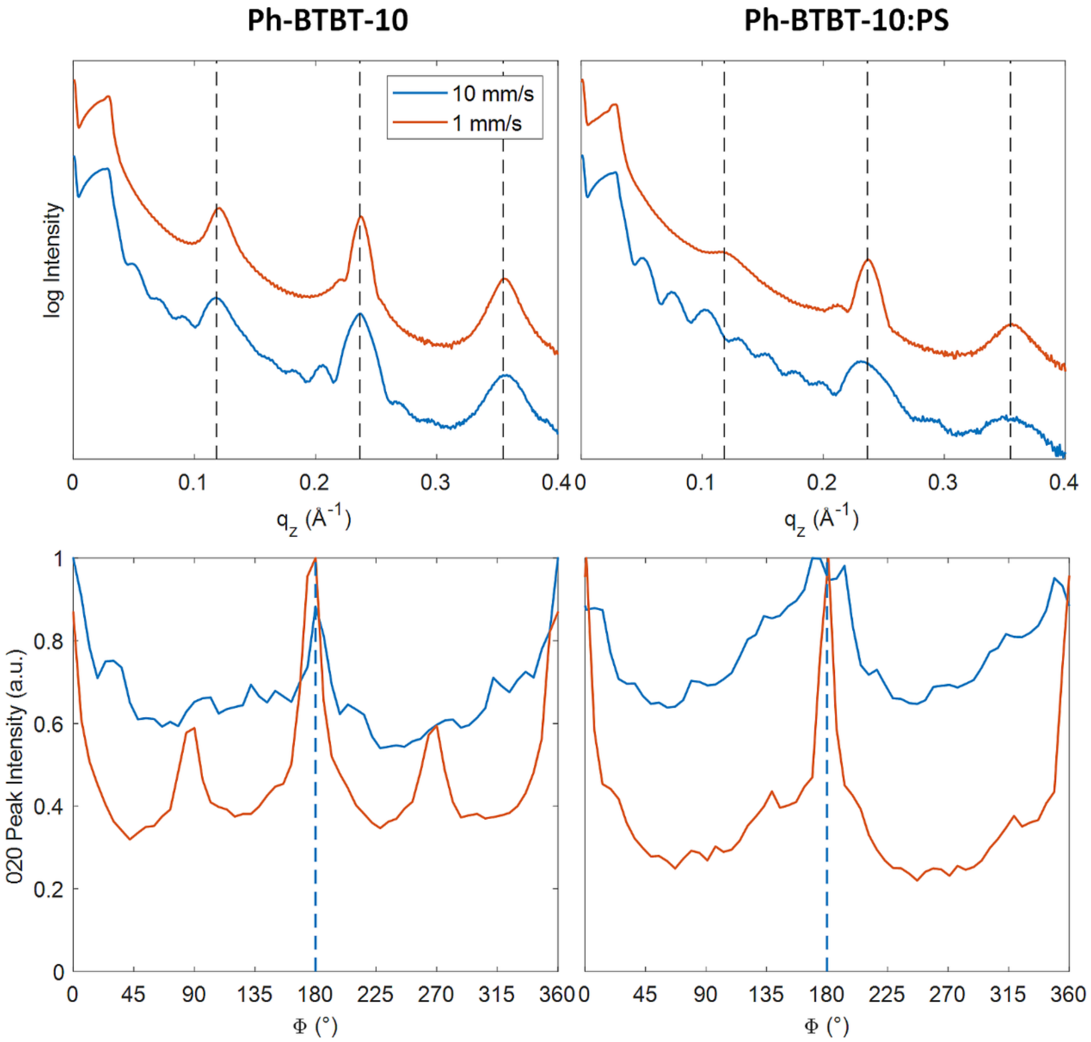

Fig. S4. X-Ray reflectivity curves of BAMS coated Ph-BTBT-10 thin films prepared from o-xylene solution without (left) and with (right) PS blend (top), Kiessig fringes reveal homogenous films with a constant film thickness of 25 nm. Rotation-dependent intensity of the 0±20-peaks from GIXD with respect to the angle measured between the coating direction axis and the conducting channel, indicated by the dashed line (bottom) for different coating speeds.

Table S2. Summary of 002 peak parameters and corresponding vertical crystal size for shear coated Ph-BTBT-10 thin films prepared from o-xylene solution, together with the in-plane mosaicity of the 020 Bragg peak.

	002 Peak Width [Å^{-1}]	Vertical Crystal Size [nm]	In-plane alignment FHWM [°]
10 mm/s Ph-BTBT-10	0.0145	43	50
1 mm/s Ph-BTBT-10	0.0091	69	18
10 mm/s Ph-BTBT-10:PS	0.0260	24	46
1 mm/s Ph-BTBT-10:PS	0.0123	51	16

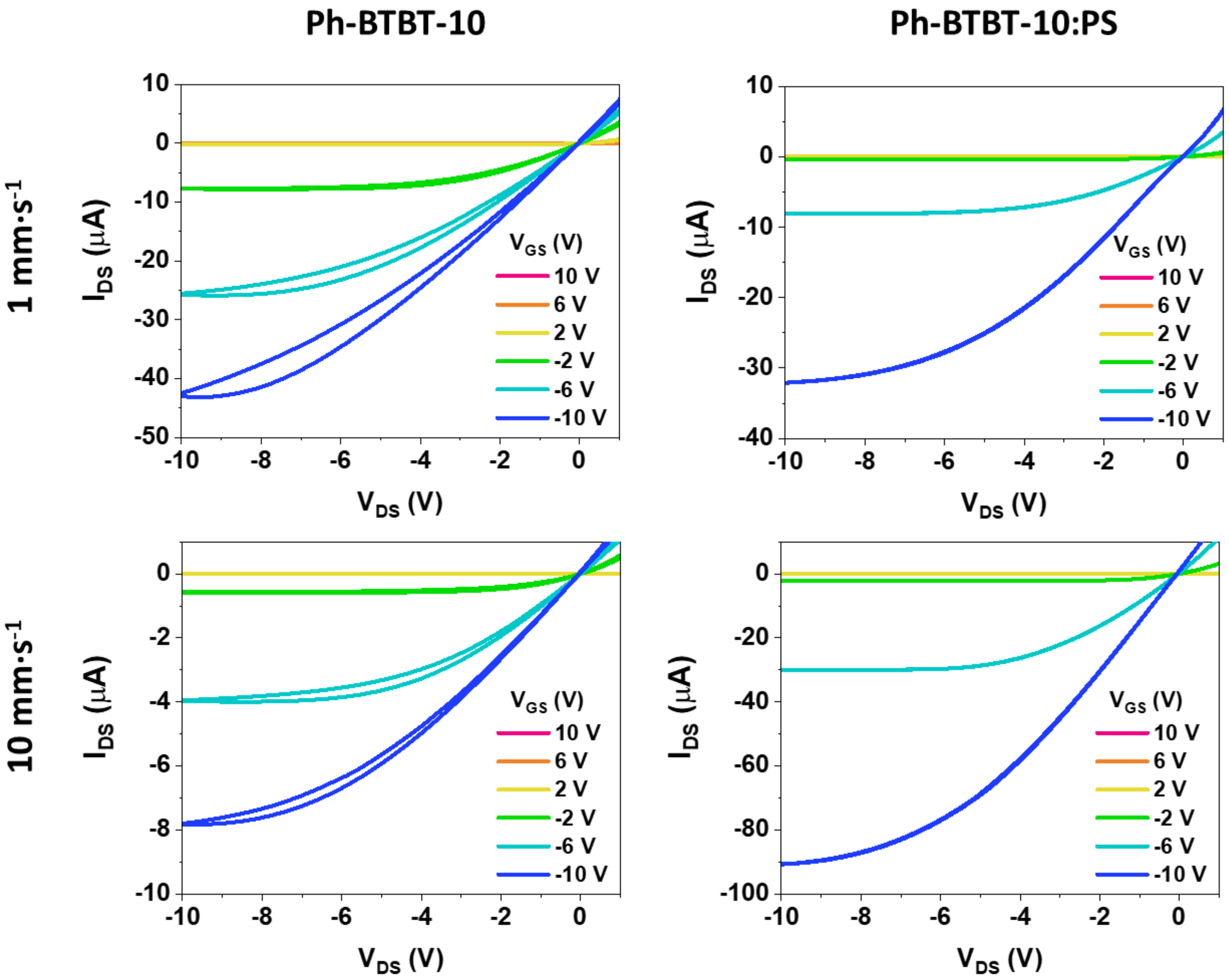

Fig. S5. Output characteristics of the OFETs based on Ph-BTBT-10 and Ph-BTBT-10:PS deposited from PhCl solutions at 1 mm/s and 10 mm/s.

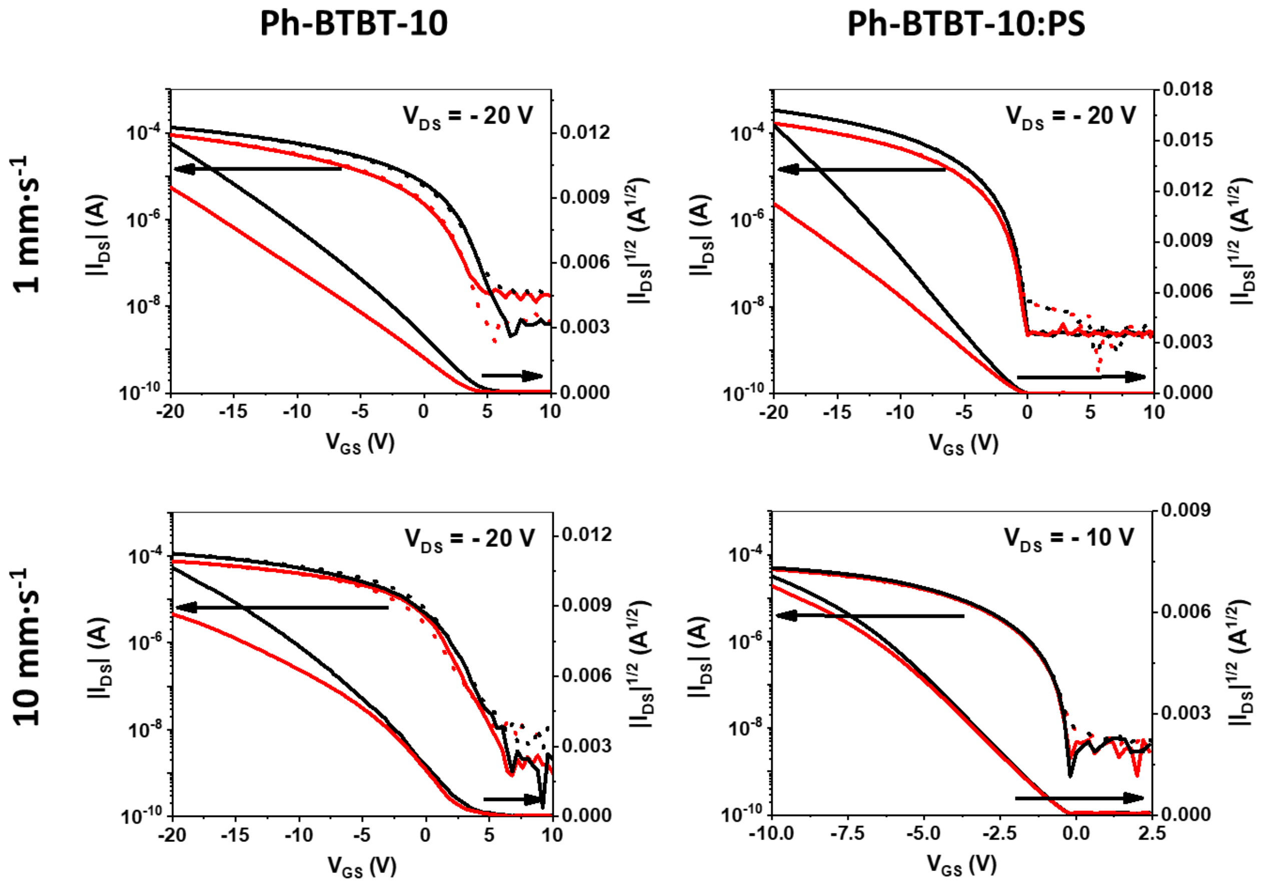

Fig. S6. Transfer characteristics in the saturation regime of typical Ph-BTBT-10 and Ph-BTBT-10:PS films prepared by BAMS from o-xylene solutions at 1 and 10 mm/s. Continuous lines correspond to forward, while dotted lines correspond to reverse sweeps of gate voltages. Black lines correspond to devices with the channel length (L) parallel to the coating direction and red lines correspond to the perpendicular ones.

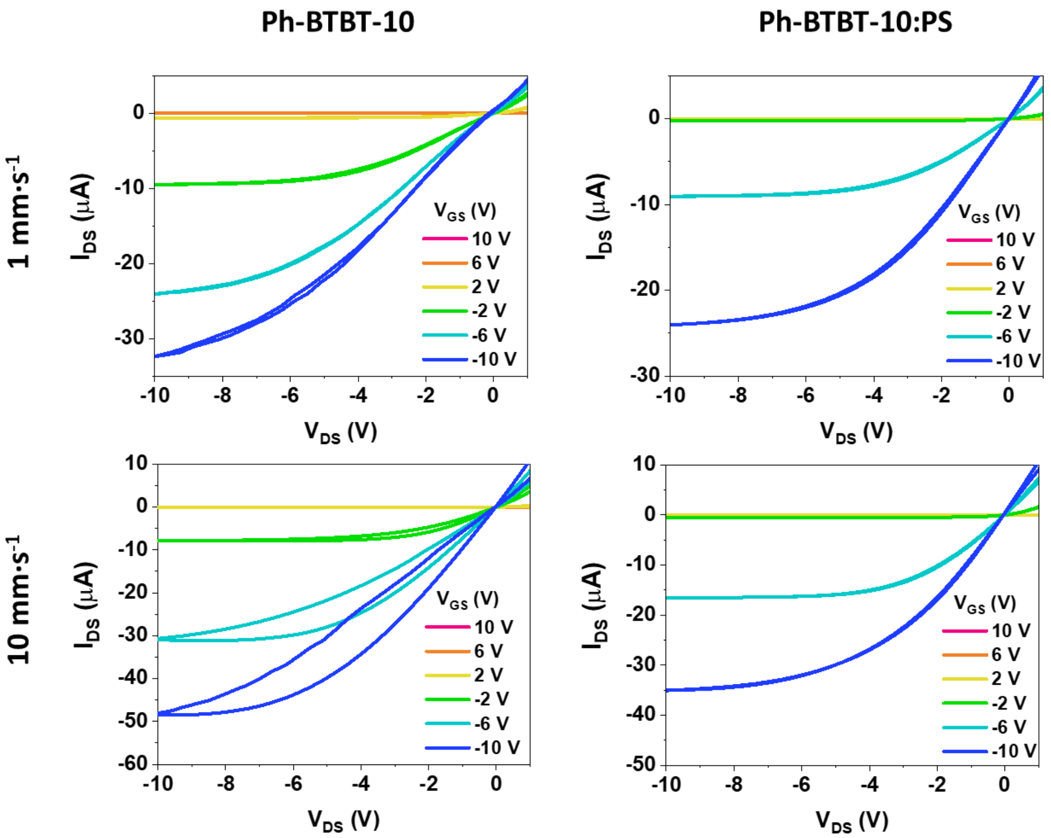

Fig. S7. Output characteristics of the OFETs based on Ph-BTBT-10 and Ph-BTBT-10:PS deposited from o-xylene solutions at 1 mm/s and 10 mm/s.

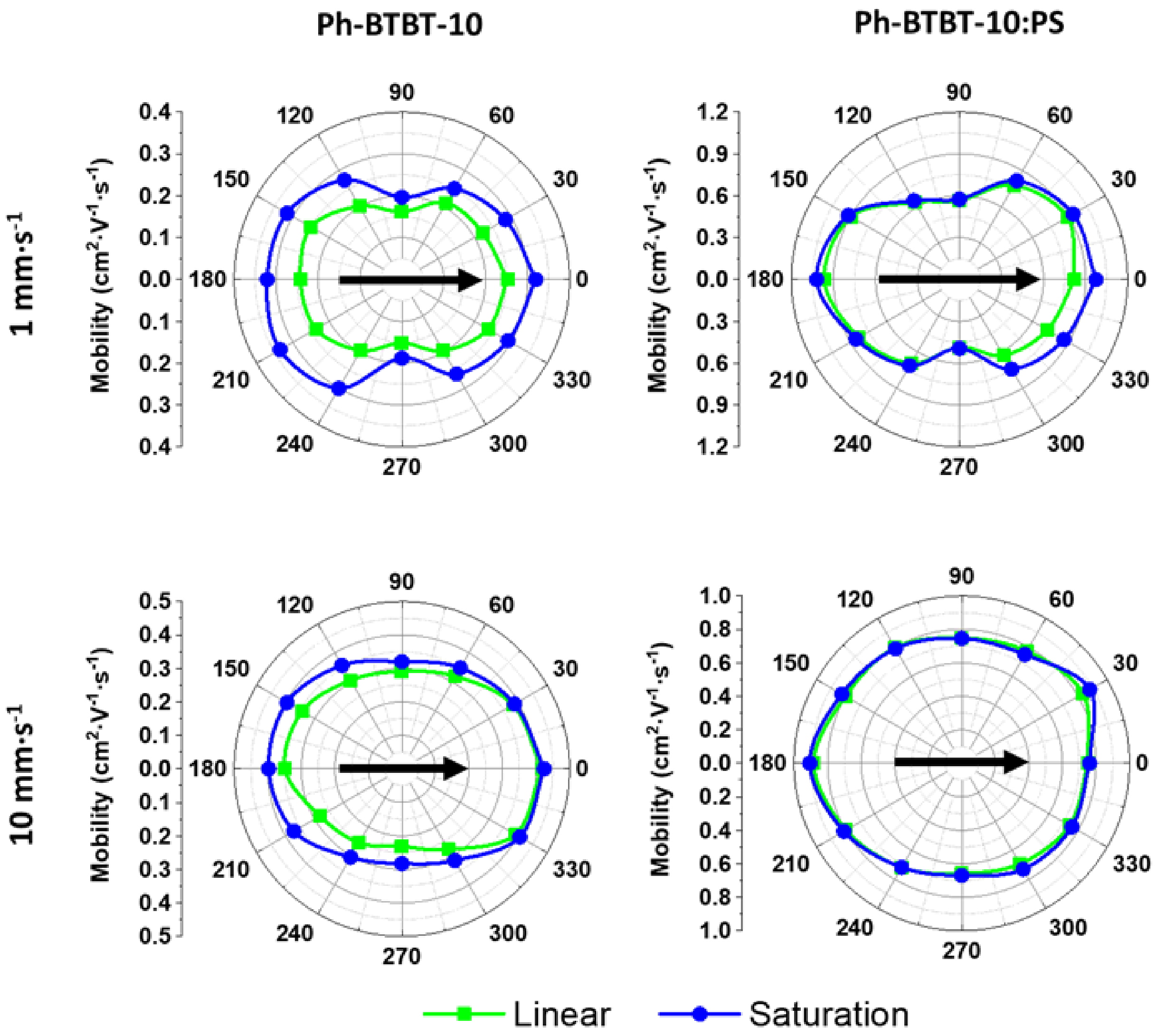

Fig. S8. Polar plot of the linear and saturation mobility of the Ph-BTBT-10 and Ph-BTBT-10:PS films prepared from o-xylene solutions at low and high coating speed (angle of the conducting channel L with respect to the coating direction). The direction of the solution shearing is indicated with a black arrow and the L of the devices were 100 µm.

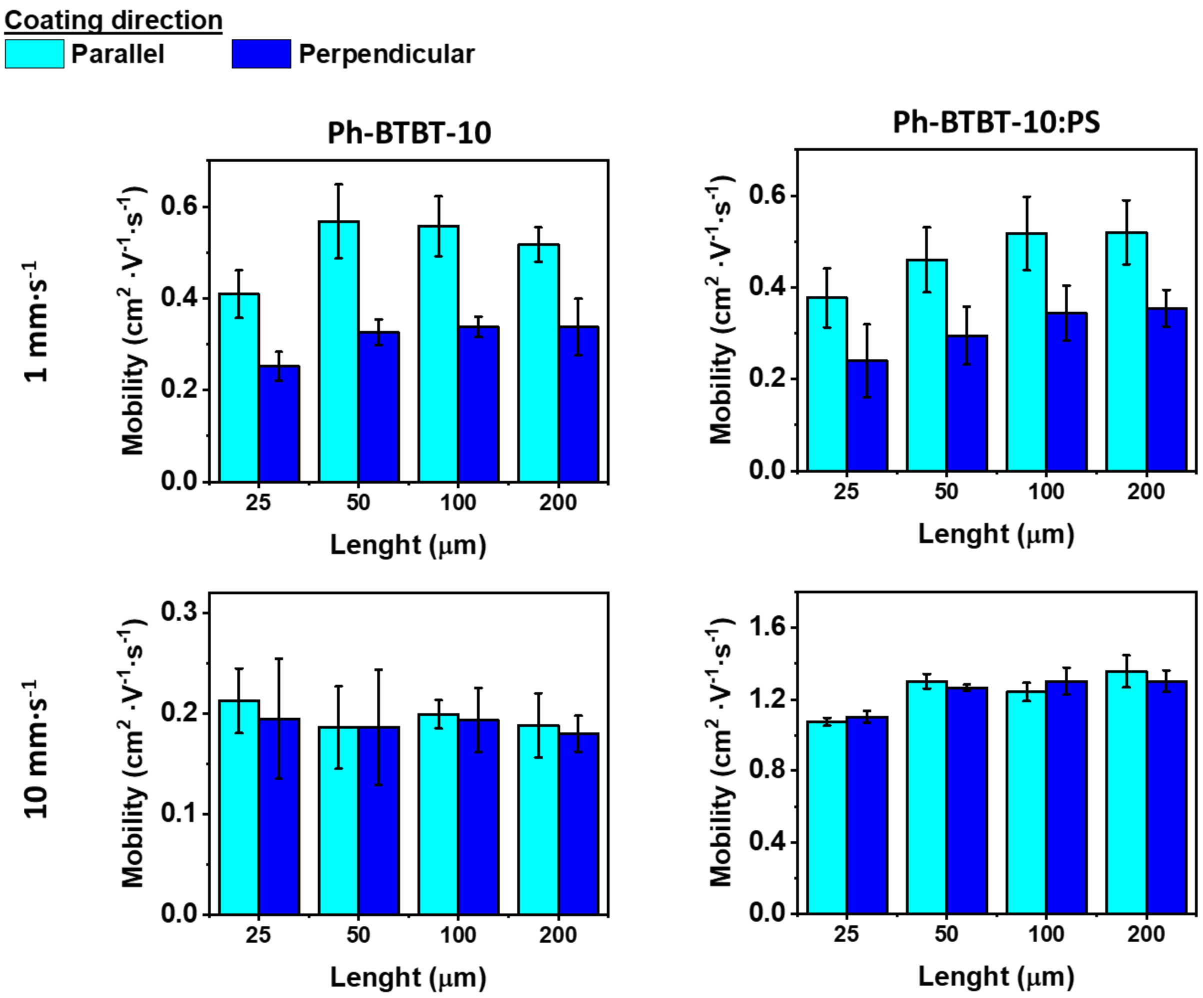

Fig. S9. Bar graph comparing the saturation mobility of Ph-BTBT-10 and Ph-BTBT-10:PS films in parallel and perpendicular orientations with respect to the coating speed. The films were prepared from chlorobenzene solutions at low and high coating speed.
